# Fertility and Neonatal Outcomes of Freeze-All vs. Fresh Embryo Transfer in Women With Advanced Endometriosis

**DOI:** 10.3389/fendo.2019.00770

**Published:** 2019-11-08

**Authors:** JIayi Wu, Xiaoyan Yang, Jiaan Huang, Yanping Kuang, Yun Wang

**Affiliations:** Department of Assisted Reproduction, Shanghai Ninth People's Hospital, Shanghai Jiao Tong University School of Medicine, Shanghai, China

**Keywords:** freeze-all strategy, frozen–thawed embryo transfer, advanced endometriosis, pregnancy outcome, neonatal outcome

## Abstract

**Background:** Eutopic endometrium from women with endometriosis has functional changes in several aspects, which may largely account for the decrease in the quality of endometrial receptivity. It is of utmost importance to know whether freeze-all strategy can restore optimal receptivity in endometriotic women leading to the better ART outcomes.

**Methods:** Retrospective study involved patients with advanced endometriosis undergoing first embryo transfer cycles during the period from March 2006 to March 2017 at a tertiary care center. After propensity score matching, there were 506 women in the freeze-all group and 255 women in fresh group. Our main outcomes included the rates of implantation, clinical pregnancy, and live birth. Subgroup analyses were performed after stratification by the number of oocytes retrieved and fertilization method. Neonatal outcomes included gestational age and birth weight z-score for singletons and multiple births.

**Results:** In our matched cohort, the implantation, clinical pregnancy and live birth rates were statistically significantly higher in the freeze-all group compared with fresh transfer groups (34.4 vs. 25.5%, 51.8 vs. 38.8%, and 45.3 vs. 31.8%, all *P* < 0.001, respectively). A more beneficial effect of freeze-all cycles was found in patients who got more than 15 oocytes. Additionally, when ICSI insemination techniques were used to achieve fertilization, the advantage of freeze-all strategy was not obvious. Assessment of 382 babies showed no statistically significant difference in the mode of delivery, sex of live-born, gestational age, unadjusted median birth weight, and z-score between two study groups.

**Conclusion:** Freeze-all strategy is an attractive option to improve the outcomes of ART for women with advanced endometriosis.

## Introduction

The application of *in vitro* fertilization (IVF) began 40 years ago. More than 5 million children have been born since then who simply could not have been without the development of IVF (1). With the accumulation of clinical experience and advancement of cryopreservation techniques, frozen embryo transfer (FET) has been widely used because of the higher pregnancy rate it resulted in and a trend for lower risks of preterm birth, low birth weight, and perinatal death compared to fresh embryo transfer (ET) (2–4).

Recently, a new strategy called “freeze-all” emerged in clinical treatment, which was confirmed to further improve the outcomes of IVF (5). By cryopreserving all embryos, ovarian hyper-stimulation syndrome (OHSS) in high-risk women could be initially avoided and a better embryo-endometrium synchrony achieved with endometrium preparation could provide a physiological environment for embryo implantation. Several observational studies showed the higher rates of pregnancy and live birth and better perinatal outcomes with the freeze-all strategy (4, 6, 7). A randomized controlled trial assigned 1,508 women with the polycystic ovary syndrome (PCOS) suggested that freeze-all strategy led to a higher live birth rate (49.3% vs. 42.0%; relative risk, 1.17; 95% CI, 1.05–1.31) and a lower pregnancy loss rate (2.0% vs. 32.7%; relative risk, 0.67; 95% CI, 0.54–0.83) than did fresh-embryo transfer (8). Another multicenter, randomized, controlled trial involving 2,157 ovulatory women indicated the risk of the OHSS in freeze-all group was significantly lower than that in fresh embryo transfer group (0.6% vs. 2.0%; relative risk, 0.32; 95% CI, 0.14–0.74) (9). As none of these studies were conducted in advanced endometriosis patients, the effect of freeze-all strategy in this aspect was still unknown. Only two studies on endometriosis-related infertility showed that freeze-all policy caused better pregnancy outcomes but with small sample sizes (10, 11). Additionally, the outcome of infants was lacking. Of note, previous study proved women with advanced endometriosis had lower rates of implantation, cumulative pregnancy, and live-birth compared with patients with mild endometriosis or tubal factor infertility (12). And to date, the best practice for treating stage III/IV endometriosis-related infertility is still a matter of debate in the medical community. It is extremely important to determine if there are any differences between the two procedures in fertility and neonatal outcomes.

Consequently, the aim of the present study is to assess the influence of freeze-all strategy, as compared with fresh embryo transfer, on pregnancy and neonatal outcomes in women with advanced endometriosis.

## Materials and Methods

### Study Design

The data in this study were obtained from the ART database at the Department of Assisted Reproduction of the Shanghai Ninth People's Hospital, affiliated with the Jiao Tong University School of Medicine between March 2006 and March 2017. Our study was approved by the hospital's Ethics Committee (Institutional Review Board). The following women were included: (1) diagnosed with endometriosis by laparoscopy and classified stage III to IV according to the revised American Society for Reproductive Medicine (ASRM) scoring system (13); (2) scheduled for either a fresh or a frozen embryo transfer on day 3 (cleavage-stage) in both IVF and ICSI cycles; (3) follow-up available up to the end of pregnancy. Exclusion criteria included: (1) simultaneous transfer of embryos from different ovarian stimulation cycles; (2) diagnosis of polycystic ovary syndrome based on the modified Rotterdam diagnostic criteria; (3) diagnosis of intrauterine disease including endometrial polyps or submucosal myomas, as determined by ultrasound or hysteroscopy. Only the first embryo transfer cycles of patients were included for analysis.

In our center, embryo development was evaluated on day 3, and only high-quality cleavage-stage embryos [at least six blastomeres with ≤20% fragmentation according to the Cummins' Criteria (14)] were selected for transfer. In fresh group, all patients were scheduled for a day-3 fresh ET at first, and supernumerary embryos were vitrified. While in the freeze-all group, the entire cohort of good-quality embryos was vitrified on day 3.

There is growing clinical and scientific evidence showing that FET improves outcomes for both the mother and baby, and the major reason currently given for continuing with fresh embryo transfers are patient preference and governmental funding models that discourage a freeze-all strategy as stated by Evans et al. (6). The freeze-all strategy has been widely used due to its great advantages (15), and so, many patients receive treatment with all embryos cryopreserved, especially for women with premature P elevation and ovarian hyperstimulation syndrome (OHSS) risk. Notably, the couples received careful counseling regarding protocol of FET and fresh ET. Some couples may consider factors such as the risk in embryo damage after cryopreservation, longer duration of a cycle and more visits to the hospital in the FET cycles. The couples' opinion regarding transfer performed in fresh or frozen cycle was taken into consideration. Therefore, the final decision between freeze-all strategy and fresh embryo transfer for the first attempt was based on a joint decision by the patient and the doctor.

### Statistical Analysis

#### Propensity Score Matching Procedures

Before propensity score matching, the baseline characteristics distributed between freeze-all and fresh group were not the same. In order to account for this imbalance, a propensity score was generated using a logistic regression model as suggested by Rosenbaum and Rubin (16, 17). A total of 16 covariates were selected for the propensity score model as follows: age, BMI, duration of infertility, gravidity, parity, endocrinological profile (basal FSH, LH, E_2_, P, and AFC), concomitant infertility factors (tubal factors and male factors), procedure, number of oocytes retrieved, number of embryos available, number of embryos transferred. Following propensity score generation, patients were matched by using 1:2 nearest neighbor matching without replacement. Cramer's V and R^2^ were used to assess the balance in baseline covariates for categorical variables and continuous variables, respectively, where values closer to zero represent more balance between two groups.

#### Outcomes

Our main outcomes included the rates of implantation, clinical pregnancy, and live birth. The implantation rate was calculated as the number of gestational sacs divided by the number of embryos transferred. Clinical pregnancy was defined as the presence of a gestational sac with fetal heart activity in the ultrasound examination. It includes ectopic pregnancy. A miscarriage was defined as the pregnancy loss before 12 weeks of gestation. Live birth was defined as the number of women delivered at least one live neonate. Additionally, subgroup analyses were performed after stratification by the number of oocytes retrieved and fertilization method.

Gestational age was counted from the day of embryo transfer, defined as day 17 of the menstrual cycle. Preterm delivery (delivery at <37 weeks of gestation), very preterm delivery (delivery at <32 weeks of gestation),LBW (birth weight <2,500 g), very LBW (birth weight <1,500 g), term LBW (LBW at 37 weeks of gestation or greater) and preterm LBW (LBW at <37 weeks of gestation) were all counted. Additionally, we calculated a birth weight z-score (18) for each child using the following formula: (weight of individual case at given gestational age—mean weight of reference population at same gestational age)/standard deviation in the reference population, in order to adequately account for the effect of gestational age and newborn gender on birth weight. Two reference populations were used to calculate the birth weight z-scores for singletons (19) and multiple births (20) respectively.

#### Other Analyses

The chi-squared test or Fisher's exact test was used for categorical comparisons as appropriate. The Mann–Whitney–Wilcoxon test was used for continuous variables. The significant difference was considered at *P*-value <0.05. Data were carried out using the Statistical Package for the Social Sciences software (version 23.0, SPSS Inc., Chicago, USA).

## Result

**Figure 1:**
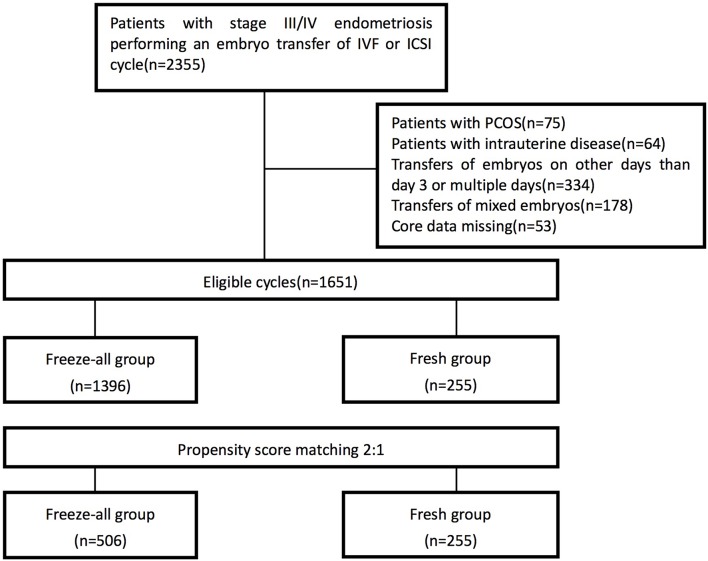
Flow chart.

A total of 1,651 women were extracted from the database in Shanghai Ninth People's Hospital affiliated with Shanghai Jiao Tong University School. Patient distribution before and after propensity score matching is shown in Figure 1. After matching, there were 506 women in the freeze-all group and 255 women in fresh group. Main maternal and treatment characteristics of the population before and after propensity score matching are summarized in Table 1. After propensity score matching, all the baseline characteristics between the two study groups are balanced.

**Table 1:** Main maternal and treatment characteristics before and after matching.

	**Unmatched group**				**Propensity score matched group**			
	**Freeze-all** ** (*n* = 1396)**	**Fresh** ** (*n* = 255)**	R2^*^	**Cramer's V^†^**	**Freeze-all** ** (*n* = 506)**	**Fresh** ** (*n* = 255)**	R2^*^	**Cramer's V^†^**
**Demographics**								
Age	32.0 (29.0, 35.0)	33.0 (30.0, 35.0)	0.0017		32.0 (29.0, 35.0)	33.0 (30.0, 35.0)	0.0011	
BMI	20.7 (19.2, 22.4)	20.6 (19.1, 22.3)	0.0005		20.7 (19.4, 22.3)	20.6 (19.1, 22.3)	<0.0001	
**Fertility characteristics**								
Duration of infertility	3.0 (2.0, 5.0)	4.0 (2.0, 6.0)	0.0130		3.5 (2.0, 6.0)	4.0 (2.0, 6.0)	0.0011	
Gravidity	0.0 (0.0, 1.0)	0.0 (0.0, 1.0)	0.0005		0.0 (0.0, 1.0)	0.0 (0.0, 1.0)	0.0009	
Parity	0.0 (0.0, 0.0)	0.0 (0.0, 0.0)	0.0026		0.0 (0.0, 0.0)	0.0 (0.0, 0.0)	<0.0001	
**Concomitant infertility factors**								
Tubal factors	824 (59.0)	132 (51.8)		0.0531	269 (53.2)	132 (51.8)		0.0132
Male factors	227 (16.3)	52 (20.4)		0.0398	109 (21.5)	52 (20.4)		0.0131
**Endocrinological profile**								
Basal FSH (IU/L)	5.9 (5.0, 7.3)	5.2 (3.8, 7.0)	0.0066		5.6 (4.8, 6.7)	5.2 (3.8, 7.0)	0.0016	
Basal LH (IU/L)	3.3 (2.4, 4.4)	2.2 (1.2, 3.9)	0.0002		3.2 (2.4, 4.3)	2.2 (1.2, 3.9)	0.0006	
Basal E2 (pg/mL)	36.0 (26.0, 49.0)	31.0 (17.0, 49.0)	0.0003		36.0 (27.0, 49.3)	31.0 (17.0, 49.0)	<0.0001	
Basal P (ng/mL)	0.3 (0.2, 0.4)	0.2 (0.2, 0.4)	0.0009		0.3 (0.2, 0.4)	0.2 (0.2, 0.4)	0.0008	
Antral follicle count	8.0 (5.0, 11.0)	9.0 (6.0, 10.0)	0.0002		9.0 (5.0, 12.0)	9.0 (6.0, 10.0)	<0.0001	
**Procedure**								
IVF	1000 (71.6)	190 (74.5)		0.0447	359 (70.9)	190 (74.5)		0.0378
ICSI	292 (20.9)	54 (21.2)			121 (23.9)	54 (21.2)		
IVF+ICSI	104 (7.4)	11 (4.3)			26 (5.1)	11 (4.3)		
No. of oocytes retrieved	7.0 (4.0, 12.0)	8.0 (5.0, 13.0)	0.0041		8.0 (4.0, 13.0)	8.0 (5.0, 13.0)	0.0003	
No. of embryos available	3.0 (2.0, 5.0)	4.0 (2.0, 6.0)	0.0078		4.0 (2.0, 6.0)	4.0 (2.0, 6.0)	0.0002	
No. of embryos transferred	2.0 (2.0, 2.0)	2.0 (2.0, 2.0)	0.0029		2.0 (2.0, 2.0)	2.0 (2.0, 2.0)	0.0007	

*Continuous data are presented as median (interquartile range). Categorical data are presented as n (%)*.

* *R^2^used to assess balance in covariates for continuous variables*.

† *Cramer's V used to assess balance in covariates for categorical variables*.

In our matched cohort (Table 2), the implantation, clinical pregnancy and live birth rates were statistically significantly higher in the freeze-all group compared with fresh transfer groups, with OR 1.60 (95% CI, 1.26–2.02; *P* < 0.001) for implantation, OR 1.69 (95% CI, 1.25–2.30; *P* < 0.001) for clinical pregnancy, and OR 1.74 (95% CI, 1.27–2.39; *P* < 0.001) for live birth. No remarkable differences emerged in the rates of biochemical pregnancy, miscarriage, ectopic pregnancy, and multiple births between groups.

**Table 2:** Main pregnancy characteristics.

	**Freeze-all** ** (*n* = 506)**	**Fresh** ** (*n* = 255)**	***P*-value**	**OR** ** (95% CI)**
Implantation rate	34.4 (332/964)	25.5 (125/491)	<0.001	1.60 (1.26–2.02)
Biochemical pregnancy rate	7.1 (36/506)	6.7 (17/255)	0.813	1.07 (0.59–1.95)
Clinical pregnancy rate	51.8 (262/506)	38.8 (99/255)	<0.001	1.69 (1.25–2.30)
Multiple pregnancy rate	21.0 (55/262)	18.2 (18/99)	0.553	1.20 (0.66–2.16)
Miscarriage rate	12.2 (32/262)	16.2 (16/99)	0.326	0.72 (0.38–1.38)
Ectopic pregnancy rate	0.4 (1/262)	1.0 (1/99)	0.490	0.38 (0.02–6.06)
Live birth rate	45.3 (229/506)	31.8 (82/255)	<0.001	1.74 (1.27–2.39)

*Data are presented as % (n/N). OR, odds ratio; CI, confidence interval*.

Two subgroup analyses were then performed using the propensity matched data set to isolate the effect of the freeze-all strategy. First, patients were stratified based on the number of oocytes retrieved (Table 3). The difference in pregnancy outcomes between fresh and freeze-all cycles was not significant in low responders (1–5 OR). However, when women got more than 15 oocytes, the rates of clinical pregnancy (61.6% vs. 21.7%; OR 5.79; 95% CI, 2.49–13.46, *P* < 0.001) and live birth (57.5% vs. 21.7%; OR 4.88; 95% CI, 2.10–11.30, *P* < 0.001) were great higher in freeze-all group than in fresh group. Next, the data were analyzed according to the fertilization method. Considering the limited number of IVF+ICSI cycles in our study, we put the ICSI and IVF+ICSI cycles in the same group for analysis. As shown in Table 4, CPR and LBR in IVF cycles still significantly differed between the study groups (*P* < 0.001). For cycles performed with ICSI (including ICSI and IVF+ICSI cycles), there were no statistically significant differences in the selected pregnancy outcomes between freeze-all and fresh groups.

**Table 3:** Pregnancy outcomes according to the number of oocytes retrieved.

	**Freeze-all**	**Fresh**	**OR (95% CI)**	***P***
1–5 (*n* = 237)				
CPR	75 (44.4)	25 (36.8)	1.37 (0.77–2.45)	0.284
LBR	61 (36.1)	21 (30.9)	1.26 (0.69–2.31)	0.446
5–10 (*n* = 243)				
CPR	83 (55.0)	44 (47.8)	1.33 (0.79–2.24)	0.280
LBR	73 (48.3)	36 (39.1)	1.46 (0.86–2.47)	0.162
11–15 (*n* = 162)				
CPR	59 (52.2)	20 (40.8)	1.58 (0.80–3.12)	0.184
LBR	53 (46.9)	15 (30.6)	2.00 (0.98–4.08)	0.056
>15 (*n* = 119)				
CPR	45 (61.6)	10 (21.7)	5.79 (2.49–13.46)	<0.001
LBR	42 (57.5)	10 (21.7)	4.88 (2.10–11.30)	<0.001

*Data were presented as n (%). CPR, clinical pregnancy rate; LBR, live birth rate; OR, odds ratio; CI, confidence interval*.

**Table 4:** Pregnancy outcomes according to the fertilization method.

	**Freeze-all**	**Fresh**	**OR (95% CI)**	***P***
IVF (*n* = 549)				
CPR	193 (53.8)	72 (37.9)	1.91 (1.33–2.73)	0.0004
LBR	171 (47.6)	57 (30.0)	2.12 (1.46–3.08)	<0.0001
ICSI^*^(*n* = 212)				
CPR	69 (46.9)	27 (41.5)	1.25 (0.69–2.25)	0.467
LBR	58 (39.5)	25 (38.5)	1.04 (0.57–1.90)	0.891

*Data were presented as n (%). CPR, clinical pregnancy rate; LBR, live birth rate; OR, odds ratio; CI, confidence interval*.

* *Including both ICSI and IVF+ICSI cycles*.

Neonatal outcomes are shown in Table 5. A total of 382 infants (excluded for 2 vanishing twins) were delivered following 761 embryo transfer cycles, including 236 singletons and 146 multiple births. For all these babies, the mode of delivery, sex of live-born, gestational age, unadjusted median birth weight, z-score, PTD, VPTD, LBW, VLBW, term LBW, and preterm LBW did not differ significantly in two groups.

**Table 5:** Main neonatal outcomes.

	Singletons^*^			**Multiple births**		
	**Freeze-all (*n* = 172)**	**Fresh (*n* = 64)**	**P value**	**Freeze-all (*n* = 110)**	**Fresh (*n* = 36)**	***P*-value**
Cesarean section	131 (76.2)	47 (73.4)	0.666	104 (94.5)	36 (100.0)	0.337
Male sex	95 (55.2)	34 (53.1)	0.772	61 (55.5)	24 (66.7)	0.236
Gestational age	275.0 (269.0, 278.0)	273.0 (266.0, 279.0)	0.861	259.0 (252.0, 262.0)	257.5 (254.0, 261.0)	0.319
PTD	15 (8.7)	5 (7.8)	0.824	48 (43.6)	22 (61.1)	0.068
VPTD	2 (1.2)	0 (0.0)	1.000	4 (3.6)	2 (5.6)	0.637
Birth weight	3350.0 (3022.5, 3700.0)	3250.0 (3000.0, 3500.0)	0.272	2615.0 (2200.0, 2881.3)	2600.0 (2382.5, 2765.0)	0.966
Z-score	0.03 (−0.54, 0.71)	−0.30 (−0.72, 0.25)	0.186	−0.03 (−0.76, 0.66)	0.07 (−0.62, 0.74)	0.303
LBW	10 (5.8)	2 (3.1)	0.521	44 (40.0)	10 (27.8)	0.187
VLBW	1 (0.6)	0 (0.0)	1.000	5 (4.5)	2 (5.6)	1.000
Term LBW	2 (1.2)	0 (0.0)	1.000	13 (11.8)	4 (11.1)	1.000
Preterm LBW	8 (4.7)	2 (3.1)	0.733	35 (31.8)	8 (22.2)	0.273

* *Excluded for 2 vanishing twins*.

*Continuous data (gestational age, birth weight, and z-score) are presented as median (interquartile range). Categorical data (all other variables) are presented as n (%)*.

## Discussion

The present study showed that freeze-all strategy led to a significantly higher implantation rate, clinical pregnancy rate and live birth rate compared to fresh ET transfer. Additionally, there was no difference in the outcomes of birth weight or gestational age at delivery when comparing two study groups. To our knowledge, this is the first large retrospective study to compare the fertility and neonatal outcomes of the first embryo transfer cycles following freeze-all strategy vs. fresh transfer in patients with advanced endometriosis.

To date, only two studies were identified to explore the effect of freeze-all strategy in women with endometriosis. A retrospective matched cohort study, including 135 endometriosis-affected women in the fresh ET and deferred ET group, respectively, reported significantly higher cumulative clinical pregnancy rate (43.0% vs. 29.6%) and cumulative ongoing pregnancy rate (34.8% vs. 17.8%) in the deferred ET group compared to the fresh ET group (11). The other study involving 521 IVF-ET/ICSI cycles in patients with mild endometriosis showed that the early abortion rate (6.7% vs. 14.6%) is lower and the live birth rate (33.7% vs. 28.4%) is higher in the first FET cycle after whole embryo cryopreservation (10). Nevertheless, the validity of the (21)se results is actually hampered by methodological limitations, since there were some differences persisted in baseline data between the groups, which might have biased the results. Moreover, the literature on advanced endometriosis is limited, and neither of these two studies have reported the neonatal outcomes after freeze-all strategy, which may be partly due to the small sample sizes they had.

Our study, aiming to overcome methodological shortcomings in above-mentioned studies and enrolling a large cohort of patients, demonstrated that for women with advanced endometriosis received freeze-all strategy had a higher percentage of implantation rate, clinical pregnancy rate, and live birth rate relative to fresh cycles. This result was somewhat consistent with several previous randomized controlled trials performed in women not restricted to endometriosis. Shapiro et al. (4) revealed significantly greater probability of clinical pregnancy with thawed embryos, controlled for differences in embryo quality, when compared with fresh in a cohort of 122 high responders (>15 antral follicles). A higher frequency of live birth and a lower frequency of pregnancy loss were found after freeze-all strategy in PCOS patients (8), and in ovulatory women a significantly lower risk of the OHSS was reported in the freeze-all group than in fresh group (9). Another small randomized controlled trial comparing freeze-all with fresh embryo transfer after preimplantation genetic screening showed the ongoing pregnancy rate (80% vs. 61%) and live birth rate (77% vs. 59%) in the freeze-all group were significantly higher compared with the fresh group (22).

In the subgroup analysis we found the advantages of freeze-all strategy were becoming more and more significant as the number of oocytes retrieved increased. This result was close to several previous studies. Higher rates of implantation and ongoing pregnancy were reported in freeze-all group than fresh ET group among women with 10–15 oocytes retrieved (OR) but not among those with 4 to 9 oocytes retrieved (23). A meta-analysis including five randomized controlled trials revealed that the freeze-all strategy could be favorable when high numbers of oocytes are collected, but when the mean number of oocytes collected is <15, the freeze-all strategy does not appear to be advantageous (24). Another analysis of 82,935 patient cycles also indicated that the freeze-all strategy is beneficial only in high responders (≥15 OR) but not in intermediate (6–14 OR) or low responders (1–5 OR) (25). It is interesting to note that although present study got a similar trend as previous studies, the freeze-all policy still had advantages at each OR level compared to fresh ET for advanced endometriosis women. We speculate that the difference is due to the abnormalities of endometrium in patients with III to IV endometriosis, since they usually have a less receptive endometrium than do women with other infertility causes.

When patients were stratified based on the fertilization method, the pregnancy outcome of freeze-all IVF group was still better than that of the fresh group. While in ICSI cycles, the advantage of freeze-all strategy was not obvious. The underlying mechanism for this finding is unclear and likely complex. It is speculated that decreased potential for embryonic development may play a role. A number of previous studies have reported a reduced ability of ICSI-derived embryos to develop to the blastocyst stage *in vitro* (26–28). This phenomenon may be associated with the injection procedure rather than with the origin of the male gamete, since it has also been found in a sibling oocyte study (29). Moreover, the decreased potential for *in vitro* blastocyst formation may be magnified following cryopreservation at early cleavage stages (30). Therefore, it is not surprising to see that the advantage of FET relative to fresh embryo transfer is limited when ICSI insemination techniques are used to achieve fertilization.

Several studies suggested that birth weight of live born may be higher after FET than those after fresh ET for all infertility women (31, 32). Notably, this difference only appeared in autologous cycles but not in donated cycles (33, 34), suggesting that unfriendly uterine environment before ET may be the main factor contributing to the adverse outcome in fresh cycles. Moreover, a retrospective study showed the incidence of low birth weight increased with increasing number of oocytes retrieved in either fresh or frozen group, which suggested that the supraphysiologic hormonal milieu at the time of oocyte retrieval may also affect the quality of embryos (25). Nevertheless, in line with our present results, a randomized controlled trial performed in ovulatory patients showed that there was no significant difference in birth weight between freeze-all and fresh groups (9).

The endometrium of women with endometriosis differs from the endometrium of healthy unaffected women (35), which may largely account for the decrease in the quality of endometrial receptivity. Therefore, it is of utmost importance to choose the appropriate strategy of embryo transfer, especially for women with advanced endometriosis. A number of studies have reported the impact of ovarian stimulation in fresh ET cycles on the early peri-implantation uterine environment. Firstly, decreased endometrial and subendometrial blood flow have been found in stimulated cycles compared to natural cycles as measured by three-dimensional power Doppler ultrasound (36). Moreover, some histopathologic changes of the stimulated endometrium have been confirmed, including advancement of endometrial maturation (37) and premature formation of nucleolar channel systems (38). In addition, during fresh embryo transfer cycles, the disruptions in the transcriptional activity of genes involved in endometrial receptivity have been proved by several researchers (39–41). The aforementioned changes are associated with the hyperestrogenic milieu generated during fresh IVF, which can in turn impair early embryonic adhesion (42), and, therefore, the implantation potential of embryos. Thus, it is not surprising that in present study the implantation, clinical pregnancy and live birth rates in fresh cycles were lower than rates in freeze-all cycles.

## Strengths and Limitations

A main strength of our study is that it included 761 patients with advanced endometriosis over 11 years, which is the largest number study on this topic to-date, offering an opportunity to compare the neonatal outcomes between two embryo transfer strategies. Moreover, we adopted the propensity score matching method, presenting some methodological similarities with randomized controlled trials (43), to ensure that the two groups are similar in terms of patient characteristics and make outcomes under the matched groups independent of treatment assignment (44). In addition, we applied a set of rigid inclusion criteria to optimize the quality of data. In order to minimize the influence of endometriosis stages, we merely collected the data of women diagnosed with stage III/IV endometriosis in our center. Since Chinese legislation limited the proportion of blastocyst transfer cycles within 7% to control the male birth, transfer of cleavage-stage embryos remained a priority (8, 9). In this study, we further restricted the analysis to cycles performed in day 3 embryo transfer to eliminate possible effects of culture duration on ART outcomes.

There are also some limitations in this study. First, because this is a retrospective study from a single center, so a well-designed, prospective, randomized study in women with endometriosis is needed in the future. Second, data on smoking history was not available in our database. Considering that only 2.4% of women in China smoke and the rate in infertile patients may be even lower (45), smoking is not supposed to play a role in the results because of the limited smokers within the study population.

In conclusion, we found that the first transfer in freeze-all strategy resulted in significantly higher rates of implantation, clinical pregnancy and live birth compared with fresh transfer, and the neonatal outcomes were similar between groups, suggesting that freeze-all strategy is an attractive option to improve the outcomes of ART for women with advanced endometriosis.

## Data Availability Statement

All datasets generated for this study are included in the article.

## Ethics Statement

The present work was a retrospective analysis of a study performed at the Department of Assisted Reproduction of Shanghai Ninth People's Hospital affiliated with Shanghai Jiao Tong University School of Medicine. Our study protocol was approved by the hospital's Ethics Committee (Institutional Review Board).

## Author Contributions

YW supervised the entire study, including the procedures, conception, design and completion, participated in the interpretation of the study data, and in revisions to the article. JH, XY, and YK were responsible for the collection of data. JW contributed the data analysis and drafted the article.

### Conflict of Interest

The authors declare that the research was conducted in the absence of any commercial or financial relationships that could be construed as a potential conflict of interest.

## References

[1] Kissin DM Jamieson DJ Barfield WD. Monitoring health outcomes of assisted reproductive technology. N Engl J Med. (2014) 371:91–3. 10.1056/NEJMc1404371 24988584 PMC4607032

[2] Kansal Kalra S Ratcliffe SJ Milman L Gracia CR Coutifaris C Barnhart KT. Perinatal morbidity after *in vitro* fertilization is lower with frozen embryo transfer. Fertil Steril. (2011) 95:548–53. 10.1016/j.fertnstert.2010.05.049 20663500 PMC5830094

[3] Maheshwari A Pandey S Shetty A Hamilton M Bhattacharya S. Obstetric and perinatal outcomes in singleton pregnancies resulting from the transfer of frozen thawed versus fresh embryos generated through *in vitro* fertilization treatment: a systematic review and meta-analysis. Fertil Steril. (2012) 98:368–77. 10.1016/j.fertnstert.2012.05.019 22698643

[4] Shapiro BS Daneshmand ST Garner FC Aguirre M Hudson C Thomas S. Evidence of impaired endometrial receptivity after ovarian stimulation for *in vitro* fertilization: a prospective randomized trial comparing fresh and frozen-thawed embryo transfer in normal responders. Fertil Steril. (2011) 96:344–8. 10.1016/j.fertnstert.2011.05.050 21737072

[5] Roque M Lattes K Serra S Solà I Geber S Carreras R. Fresh embryo transfer versus frozen embryo transfer in *in vitro* fertilization cycles: a systematic review and meta-analysis. Fertil Steril. (2013) 99:156–62. 10.1016/j.fertnstert.2012.09.003 23040524

[6] Evans J Hannan NJ Edgell TA Vollenhoven BJ Lutjen PJ Osianlis T. Fresh versus frozen embryo transfer: backing clinical decisions with scientific and clinical evidence. Hum Reprod Update. (2014) 20:808–21. 10.1093/humupd/dmu027 24916455

[7] Wu MY Chung CH Pan SP Jou GC Chen MJ Chang CH. Advantages of cumulative pregnancy outcomes in freeze-all strategy in high responders - A case-control matching analysis of a large cohort. J Formos Med Assoc. (2018) 117:676–84. 10.1016/j.jfma.2018.05.011 29887128

[8] Chen ZJ Shi Y Sun Y Zhang B Liang X Cao Y. Fresh versus frozen embryos for infertility in the polycystic ovary syndrome. N Engl J Med. (2016) 375:523–33. 10.1056/NEJMoa1513873 27509101

[9] Shi Y Sun Y Hao C Zhang H Wei D Zhang Y. Transfer of fresh versus frozen embryos in ovulatory women. N Engl J Med. (2018) 378:126–36. 10.1056/NEJMoa1705334 29320646

[10] Wang F Ke X Li M Clinical outcome of first frozen-thawed embryo transfer cycle after whole embryo cryopreservation in patients with mild endometriosis. J Reprod Med. (2018) 27:248–53. 10.3969/j.issn.1004-3845.2018.03.011.

[11] Bourdon M Santulli P Maignien C Gayet V Pocate-Cheriet K Marcellin L. The deferred embryo transfer strategy improves cumulative pregnancy rates in endometriosis-related infertility: a retrospective matched cohort study. PLoS ONE. (2018) 13:e0194800. 10.1371/journal.pone.0194800 29630610 PMC5890985

[12] Kuivasaari P Hippeläinen M Anttila M Heinonen S. Effect of endometriosis on IVF/ICSI outcome: stage III/IV endometriosis worsens cumulative pregnancy and live-born rates. Hum Reprod. (2005) 20:3130–5. 10.1093/humrep/dei176 16006468

[13] Vercellini P Viganò P Somigliana E Fedele L. Endometriosis: pathogenesis and treatment. Nat Rev Endocrinol. (2014) 10:261–75. 10.1038/nrendo.2013.255 24366116

[14] Cummins JM Breen TM Harrison KL Shaw JM Wilson LM Hennessey JF. A formula for scoring human embryo growth rates in *in vitro* fertilization: its value in predicting pregnancy and in comparison with visual estimates of embryo quality. J In Vitro Fert Embryo Transf . (1986) 3:284–95. 10.1007/BF01133388 3783014

[15] Zhu Q Chen Q Wang L Lu X Lyu Q Wang Y REPLY: The ‘Big Freeze': freeze-all should not be used for everyone. Hum Reprod. (2018) 8:1579–80. 10.1093/humrep/dey219 29931333

[16] Joffe MM Rosenbaum PR. Invited commentary: propensity scores. Am J Epidemiol. (1999) 150:327–33. 10.1093/oxfordjournals.aje.a010011 10453808

[17] Rubin DB. Estimating causal effects from large data sets using propensity scores. Ann Intern Med. (1997) 127:757–63. 10.7326/0003-4819-127-8_Part_2-199710151-00064 9382394

[18] Schisterman EF Whitcomb BW Mumford SL Platt RW. Z-scores and the birthweight paradox. Paediatr Perinat Epidemiol. (2009) 23:403–13. 10.1111/j.1365-3016.2009.01054.x 19689489 PMC2742985

[19] Dobbins TA Sullivan EA Roberts CL Simpson JM. Australian national birthweight percentiles by sex and gestational age, 1998–2007. Med J Aust. (2012) 197:291–4. 10.5694/mja11.11331 22938128

[20] Li Z Umstad MP Hilder L Xu F Sullivan EA. Australian national birthweight percentiles by sex and gestational age for twins, 2001–2010. BMC Pediatrics. (2015) 15:148. 10.1186/s12887-015-0464-y 26450410 PMC4599725

[21] Glujovsky D Farquhar C Quinteiro Retamar AM Alvarez Sedo CR Blake D Cleavage stage versus blastocyst stage embryo transfer in assisted reproductive technology. Cochrane Database Syst Rev. (2016). CD002118. 10.1002/14651858.CD002118.pub5 27357126

[22] Coates A Kung A Mounts E Hesla J Bankowski B Barbieri E. Optimal euploid embryo transfer strategy, fresh versus frozen, after preimplantation genetic screening with next generation sequencing: a randomized controlled trial. Fertil Steril. (2017) 107:723–30. 10.1016/j.fertnstert.2016.12.022 28139240

[23] Roque M Valle M Guimarães F Sampaio M Geber S. Freeze-all cycle for all normal responders? J Assist Reprod Genet. (2017) 34:179–85. 10.1007/s10815-016-0834-x 27817036 PMC5306402

[24] Dieamant FC Petersen CG Mauri AL Comar V Mattila M Vagnini LD. Fresh embryos versus freeze-all embryos - transfer strategies: nuances of a meta-analysis. JBRA Assist Reprod. (2017) 21:260–72. 10.5935/1518-0557.20170048 28837037 PMC5574650

[25] Acharya KS Acharya CR Bishop K Harris B Raburn D Muasher SJ Freezing of all embryos in *in vitro* fertilization is beneficial in high responders, but not intermediate and low responders: an analysis of 82,935 cycles from the Society for Assisted Reproductive Technology registry. Fertil Steril. (2018) 110:880–7. 10.1016/j.fertnstert.2018.05.024 30139718

[26] Plachot M Belaisch-Allart J Mayenga JM Chouraqui A Tesquier L Serkine AM. Outcome of conventional IVF and ICSI on sibling oocytes in mild male factor infertility. Hum Reprod. (2002) 17:362–9. 10.1093/humrep/17.2.362 11821279

[27] Miller JE Smith TT. The effect of intracytoplasmic sperm injection and semen parameters on blastocyst development *in vitro*. Hum Reprod. (2001) 16:918–24. 10.1093/humrep/16.5.918 11331638

[28] Ménézo Y Barak Y. Comparison between day-2 embryos obtained either from ICSI or resulting from short insemination IVF: influence of maternal age. Hum Reprod. (2000) 15:1776–80. 10.1093/humrep/15.8.1776 10920102

[29] Griffiths TA Murdoch AP Herbert M. Embryonic development *in vitro* is compromised by the ICSI procedure. Hum Reprod. (2000) 15:1592–1596. 10.1093/humrep/15.7.1592 10875872

[30] Archer J Gook DA Edgar DH. Blastocyst formation and cell numbers in human frozen-thawed embryos following extended culture. Hum Reprod. (2003) 18:1669–73. 10.1093/humrep/deg319 12871880

[31] Shapiro BS Daneshmand ST Bedient CE Garner FC Comparison of birth weights in patients randomly assigned to fresh or frozen-thawed embryo transfer. Fertil Steril. (2016) 106:317–21. 10.1016/j.fertnstert.2016.03.049 27397626

[32] Maheshwari A Raja EA Bhattacharya S. Obstetric and perinatal outcomes after either fresh or thawed frozen embryo transfer: an analysis of 112,432 singleton pregnancies recorded in the Human Fertilisation and Embryology Authority anonymized dataset. Fertil Steril. (2016) 106:1703–8. 10.1016/j.fertnstert.2016.08.047 27678031

[33] Vidal M Vellvé K González-Comadran M Robles A Prat M Torné M. Perinatal outcomes in children born after fresh or frozen embryo transfer: a Catalan cohort study based on 14,262 newborns. Fertil Steril. (2017) 107:940–7. 10.1016/j.fertnstert.2017.01.021 28292612

[34] Kalra SK Ratcliffe SJ Coutifaris C Molinaro T Barnhart KT. Ovarian stimulation and low birth weight in newborns conceived through *in vitro* fertilization. Obstetr Gynecol. (2011) 118:863–71. 10.1097/AOG.0b013e31822be65f 21934450 PMC3178887

[35] Sharpe-Timms KL. Endometrial anomalies in women with endometriosis. human fertility and reproduction: the oocyte, the embryo, and the uterus. (2001) 943:131–47. 10.1111/j.1749-6632.2001.tb03797.x 11594534

[36] Ng EH Chan CC Tang OS Yeung WS Ho PC. Comparison of endometrial and subendometrial blood flow measured by three-dimensional power Doppler ultrasound between stimulated and natural cycles in the same patients. Hum Reprod. (2004) 19:2385–90. 10.1093/humrep/deh384 15319389

[37] Kolibianakis E Bourgain C Albano C Osmanagaoglu K Smitz J Van Steirteghem A. Effect of ovarian stimulation with recombinant follicle-stimulating hormone, gonadotropin releasing hormone antagonists, and human chorionic gonadotropin on endometrial maturation on the day of oocyte pick-up. Fertil Steril. (2002) 78:1025–9. 10.1016/S0015-0282(02)03323-X 12413988

[38] Zapantis G Szmyga MJ Rybak EA Meier UT. Premature formation of nucleolar channel systems indicates advanced endometrial maturation following controlled ovarian hyperstimulation. Hum Reprod. (2013) 28:3292–300. 10.1093/humrep/det358 24052503 PMC3895983

[39] Mirkin S Nikas G Hsiu JG Díaz J Oehninger S. Gene expression profiles and structural/functional features of the peri-implantation endometrium in natural and gonadotropin-stimulated cycles. J Clin Endocrinol Metab. (2004) 89:5742–52. 10.1210/jc.2004-0605 15531538

[40] Horcajadas JA Riesewijk A Polman J van Os R Pellicer A Mosselman S. Effect of controlled ovarian hyperstimulation in IVF on endometrial gene expression profiles. Mol Hum Reprod. (2005) 11:195–205. 10.1093/molehr/gah150 15695772

[41] Haouzi D Assou S Mahmoud K Tondeur S Rème T Hedon B. Gene expression profile of human endometrial receptivity: comparison between natural and stimulated cycles for the same patients. Hum Reprod. (2009) 24:1436–45. 10.1093/humrep/dep039 19246470 PMC2871799

[42] Valbuena D Martin J de Pablo JL Remohí J Pellicer A Simón C. Increasing levels of estradiol are deleterious to embryonic implantation because they directly affect the embryo. Fertil Steril. (2001) 76:962–8. 10.1016/S0015-0282(01)02018-0 11704118

[43] Feng P Zhou XH Zou QM Fan MY Li XS. Generalized propensity score for estimating the average treatment effect of multiple treatments. Stat Med. (2012) 31:681–97. 10.1002/sim.4168 21351291

[44] Austin PC Double propensity-score adjustment: a solution to design bias or bias due to incomplete matching. Stat Methods Med Res. (2017) 26:201–22. 10.1177/0962280214543508 25038071 PMC5302082

[45] Zhu J Lin S Li M Chen L Lian Y Liu P. Effect of *in vitro* culture period on birthweight of singleton newborns. Hum Reprod. (2014) 29:448–54. 10.1093/humrep/det460 24408317

